# Family matters: the role of parents in adolescent motivation and exercise promotion

**DOI:** 10.3389/fped.2025.1641507

**Published:** 2026-01-30

**Authors:** Dan Bai, Mingdong Wu, Baixia Li, Danheng Zheng, Xiaoming Liu

**Affiliations:** 1 Department of Physical Education, Quzhou University, Quzhou, China; 2 School of Physical Education, Guizhou Education University, Guizhou, China; 3 School of Sports Science and Engineering, East China University of Science and Technology, Shanghai, China; 4 School of Physical Education, Shanghai University of Sport, Shanghai, China; 5 School of Physical Education, Hunan Normal University, Changsha, China

**Keywords:** exercise behavior, exercise habits, exercise motivation, junior high school students, parents

## Abstract

**Objectives:**

This study aimed to investigate the relationship between parents' exercise habits (PEH), exercise motivation (EMO), and exercise behavior (EB) among junior high school students in Shanghai, with a particular focus on the mediating effects of different types of motivation.

**Methods:**

A cross-sectional online survey was conducted in Shanghai from February to June 2023 using purposive sampling. A total of 803 questionnaires were distributed through online platforms, and 777 valid responses were included in the final analysis. Structural Equation Modeling (SEM) was applied using SmartPLS 4.0 to examine the direct and indirect pathways among variables.

**Results:**

The results showed that PEH was positively associated with EB both directly and indirectly. Among the five types of EMO, competence motivation (CMO) and fitness motivation (FMO) were positively related to EB, whereas social motivation (SMO) was negatively related to EB. Mediation analysis indicated that CMO served as a significant positive mediator, while SMO acted as a significant negative mediator in the relationship between PEH and EB. Enjoyment motivation (EJMO) and appearance motivation (AMO) were not significantly associated with EB. In addition, PEH was positively associated with all five dimensions of EMO.

**Conclusions:**

PEH play an important role in shaping adolescents' EB through distinct motivational pathways. Autonomous motivations, particularly competence-related motivation, facilitated exercise engagement, whereas socially controlled motivation was associated with lower EB. These findings highlight the need for family- and school-based interventions that emphasize autonomy and competence support rather than social pressure to promote sustained physical activity (PA) among adolescents.

## 1 Introduction

A lack of physical activity (PA) affects adolescent bone development and functional health, increases the incidence of obesity and a range of noncommunicable chronic diseases, and may contribute to vision loss (1, 2). “National policies such as China's Sunshine Sports Campaign for hundreds of millions of students” and “The Opinions on Strengthening School Sports to Promote Students’ Physical and Mental Health and All-round Development” emphasize encouraging young people to go to the playground and form good exercise habits (3). In addition, China attaches particular importance to family sports, particularly the role of parental factors in promoting PA among young people (4). But some young people remain resistant to physical exercise, one reason is the individual psychological factors (5), and the other reason is the one-sided pursuit of higher education rates; some parents emphasize their children's cultural achievements and do not support “wasting” time on physical activities (PAs) (6), which makes it difficult to form a symbiotic synergy of physical integration. Physical inactivity and a lack of exercise are significant public health issues. To help people increase their motivation for PA and exercise, most exercise psychologists recommend shifting the decisional balance by creating a belief that there are more benefits to becoming active than barriers to overcome, boosting self-efficacy, and creating social environments that promote perceptions of autonomy, competence, and relatedness (7).

For elementary and middle school students, the family is the first place where children engage in early sports and physical awareness, skill development, and improvement. A strong sports atmosphere within the family stimulates interest in sports among infants and young children. Children can gradually develop a lifelong sports consciousness and maintain physical activities as a hobby from childhood, which plays an important role in the development of lifelong adherence to the habit of physical exercise (8). In the book “Family Sports and Health Care”, Wang Zeshan indicated that family sports are the most important activities in family life. Family sports includes: “Physical education of children and young people by parents or other elderly people in the family, PAs of family members in the family living environment, and the cooperation of family sports with PAs of family members in their respective work, study and labor units”. Family sports has a positive impact on the development of children's interests and hobbies in sports and is the foundation for the formation of lifelong sports habits in children (9).

Wilk et al. (10) indicated that parental PA was not significantly related to children's perceptions of parental support for PA. However, parents reported that support for PA had a significant positive effect on their children's perceptions of parental support for boys and girls. However, Xu et al. (11) discovered a substantial relationship between mothers’ and girls' moderate-vigorous physical activity (MVPA) and total physical activity (TPA) on weekdays. Fathers' MVPA levels were substantially associated with those of boys and girls, with the paternal influence appeared to be stronger than the maternal influence. There was no substantial relationship between the fathers' and children's TPA. Sports scientists emphasize variations in the responsibilities of mothers and fathers in encouraging children to participate in sports. Fathers often make decisions regarding their children's training and competition (12, 13); and are perceived as being more involved and influential in their children's sports participation than the mothers (14). Historically, they have been perceived to have a more significant influence on athletes than mothers. The role of mothers is more strongly associated with sacrificing their own leisure or sports activities to make their children's participation in sports enjoyable (12, 13). Sukys et al. (15) discovered correlations between PEH and children's sports participation. Data analysis of girls and boys revealed that daughters' participation in sports could be predicted by both fathers' and mothers' exercise habits, whereas sons' sports activities could only be predicted by fathers' regular PAs.

Parental influence plays a critical role in shaping adolescents' health behaviors, including their engagement in PA. Firstly, parents serve as role models, and their own engagement in regular PA often encourages similar behaviors in their children (16). When students observe their parents valuing and practicing exercise, they are more likely to develop positive attitudes and intrinsic motivation toward PA (17). This modeling effect is especially strong in early adolescence, a period marked by heightened parental influence. Secondly, parents with active exercise habits are more likely to support and facilitate their children's PA through encouragement, transportation, or participation in joint activities (18). This instrumental support not only increases the likelihood of behavioral engagement but also enhances the adolescent's sense of competence and autonomy—key components of self-determination theory (19), which underpins much of the motivation research in PA. Moreover, studies have found that PEH can influence student's types of motivation. For instance, adolescents whose parents regularly exercise are more likely to report identified and intrinsic regulation—engaging in exercise because they see its value or enjoy it—rather than external regulation driven by pressure or rewards (20).

In addition, PEH can have both direct and indirect effects on students' exercise behavior. Directly, active parents often create an environment where PA is normalized and accessible. Indirectly, parents' habits may shape students' motivational processes, which in turn mediate the relationship between parental behavior and students' actual PA engagement (20). In summary, the literature suggests that PEH have significantly relationship with students' motivation to exercise and their actual exercise behavior through a combination of modeling, support, and motivational influence. Understanding this dynamic is essential for designing effective school- and community-based interventions aimed at increasing PA among adolescents.

In the context of exercise, motivation is considered a key psychological factor influencing participation and adherence. Based on Self-Determination Theory (SDT), motivation can range from intrinsic (e.g., enjoyment, personal challenge) to extrinsic (e.g., appearance, social approval) forms (21). To assess these multidimensional aspects of motivation, this study adopted the Motives for PA Measure (MPAM-R), The MPAM-R identifies five core motivational dimensions: fitness motivation (FMO), appearance motivation (AMO), competence motivation (CMO), social motivation (SMO), and enjoyment motivation (EJMO) (21). These dimensions reflect both intrinsic and extrinsic motives that drive individuals to engage in PA. The scale has been widely used in exercise psychology research and provides a comprehensive framework for examining the underlying reasons for participation in PA. Therefore, this research also proposed the following hypotheses:

*H1*: CMO will have a positive relationship with EB.
*H2*: AMO will have a positive relationship with EB.
*H3*: EJMO will have a positive relationship with EB.
*H4*: FMO will have a positive relationship with EB.
*H5*: SMO will have a positive relationship with EB.
*H6*: PEH will have a positive relationship with CMO.
*H7*: PEH will have a positive relationship with AMO.
*H8*: PEH will have a positive relationship with EJMO.
*H9*: PEH will have a positive relationship with FMO.
*H10*: PEH will have a positive relationship with SMO.
*H11*: PEH will have a positive relationship with EB.

In this research, SDT offered a valuable framework for understanding how PEH may influence adolescents' EMO and subsequent EB. According to SDT, motivation exists along a continuum ranging from controlled to autonomous regulation. When adolescents perceive their parents as active role models, they may internalize the value of PA, which supports the development of more autonomous forms of motivation—such as engaging in exercise for health, enjoyment, or personal competence. This internalization process can fulfill the three basic psychological needs proposed by SDT: autonomy, competence, and relatedness (22). As a result, PEH may contribute indirectly to EB by shaping adolescents’ EMO in a more self-determined direction.

SDT conceptualizes motivation along a continuum ranging from controlled to autonomous regulation, including external regulation, introjected regulation, identified regulation, integrated regulation, and intrinsic motivation (22). EJMO most closely reflects intrinsic motivation, as it captures engagement in exercise for inherent pleasure and interest. CMO, which emphasizes skill development, mastery, and personal challenge, aligns primarily with intrinsic motivation and identified regulation, as individuals value exercise for its contribution to personal growth and perceived competence. FMO, which involves exercising for health and physical well-being, is conceptually closer to identified regulation, reflecting a conscious valuing and internalization of the benefits of PA. In contrast, AMO is more consistent with introjected regulation, as it is driven by internal pressures such as guilt, shame, or concerns about body image and social evaluation. SMO, which emphasizes social approval, affiliation, and external social rewards, is more strongly associated with external and introjected regulation. Based on SDT, more autonomous forms of motivation (e.g., EJMO, CMO, and FMO) are expected to show stronger and more sustainable associations with EB than more controlled forms (e.g., AMO and SMO) (23). This theoretical mapping provides a clearer rationale for examining the differential mediating roles of distinct motivational types in the relationship between PEH and adolescents' EB.

**Figure 1:**
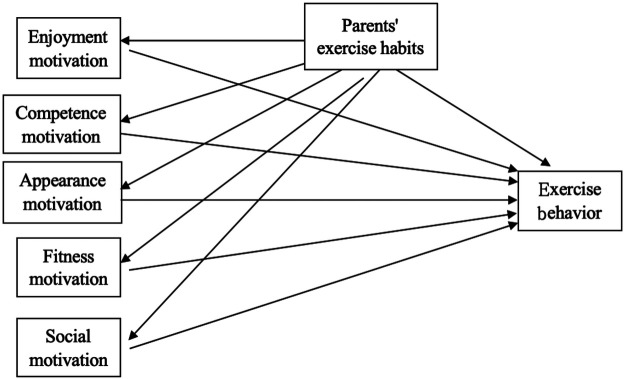
Proposed hypothetical model.

Regarding on the integrated discussion of theoretical underpinnings of the present study, Figure 1 illustrated the conceptual framework of this study. This framework indicated the mediation effect of EMO (SMO, EJMO, CMO, AMO, and FMO) on the relationship between PEH and EB in Shanghai junior high school students.

## 2 Materials and methods

### 2.1 Participants

Four representative schools were selected from different districts of Shanghai: Shanghai Minhang Huacao School, Shanghai Jingan Middle School, Shanghai Songyi Middle School, and Loushan Middle School of Minghang, Jingan, Baoshan, and Changning districts. The four selected schools belonged to middle-level schools in each district in terms of academic performance, physical fitness tests, and other related conditions. As the four schools have limited difference in the level of class for each grade, therefore, the questionnaires were selected for one class in the sixth, seventh, eighth and ninth grades of the four schools. Once the class has been finalized, we will coordinate with the class teacher. After obtaining parental consent, the survey link will be distributed in the WeChat group. Both parents and children will complete the questionnaire together. The questionnaires were distributed using stars online (a platform for collecting questionnaires in China). A total of 803 responses were received, but only 777 valid questionnaires (208 of Shanghai Minhang Huacao School, 187 of Shanghai Jingan Middle School, 195 of Shanghai Songyi Middle School and 187 of Shanghai Youai Middle School); there were 396 male and 381 female participants.

### 2.2 Exercise motivation

Exercise motivation (EMO) was assessed using “Exercise Motivation Scale” MPAM-R scale (21). To simplify the questionnaire and reduce the burden on participants, a shortened version of the MPAM-R was used in this study. Each of the five subscales (SMO, EJMO, CMO, AMO, and FMO) included 3 representative items instead of the original 6. These items were selected based on clarity and relevance for junior high school students. The questionnaire had a total of 15 questions were generated, with motivation rated from “none” to “very strong” on a scale of 1–5, with higher scores indicating higher levels of motivation. Before distributing the questionnaires, two bilingual experts specializing in PE and psychology in China evaluated the content validity and translation accuracy of the questionnaire, with all the dimension items' factor loading above 0.6, composite reliability (CR) above 0.8, the average extracted (AVE) above 0.5, and the Cronbach's alpha above 0.8, all of which indicated the questionnaires had relatively good reliability and validity.

### 2.3 Parents' exercise habits

Parents' exercise habits (PEH) were assessed using “Parents’ Exercise Habits Scale” of Dong Baolin in 2017 (24). This study believes that habits constitute automation, stability, and regularity of behavior. Three questions were primarily reflected in the three dimensions of students' exercise intensity, duration, and frequency. Question 1: “During the past few months, how often did your parents participate in physical activity regularly?”; Question 2: “How long have your parents been doing regular physical activity such as “Question 1 answer’ so far?”; Question 3: “The duration of each physical activity your parents typically participated in during the past few months”. Question 1 used a five-level scoring (1 = less than 1 time per month to 5 = at least 3 times a week); Question 2 used a five-level scoring (1 = less than one month to 5 = at least four months); Question 3 used a five-level scoring (1 = 15 min or less to 5 = at least 1 h). A higher score on the “Parents’ Exercise Habits Scale” indicates a higher parents' exercise habits level. Before distributing the questionnaires, two bilingual experts specializing in PE and psychology in China evaluated the content validity and translation accuracy of the questionnaire, with all the items' factor loading above 0.6, composite reliability (CR) 0.847, the average extracted (AVE) 0.648, and the Cronbach's alpha 0.729, all of which indicated the questionnaires had relatively good reliability and validity.

### 2.4 Exercise behavior

Exercise behavior (EB) was assessed using “Exercise Behavior Scale” of Dong Baolin in 2017 (24). This study believes exercise behavior is the purposeful use of PA as a means, and through individual cognition, motivation, decision-making, experience to achieve the behaviors required by oneself at different levels. Three questions were primarily reflected in the three dimensions of parents' exercise regularity, duration, and frequency. Question 1: “In the past month, how often did you exercise?”; Question 2: “During the last month, what was the duration of each physical activity you normally performed?”; Question 3: “In the past month, how often did you participate in physical activity in general?”. Question 1 used a five-level scoring system (1 = light exercise to 5 = high-intensity, sustained exercise with shortness of breath and profuse sweating); Question 2 used a five-level scoring system (1 = less than 10 min to 5 = more than 60 min); Question 3 used a five-level scoring system (1 = once a month or less to 5 = about 1 time a day). A higher score on the “Exercise Behavior Scale” indicates a higher exercise behavior level of junior high school students. Before distributing the questionnaires, two bilingual experts specializing in PE and psychology in China evaluated the content validity and translation accuracy of the questionnaire, with all the items' factor loading above 0.6, composite reliability (CR) 0.833, the average extracted (AVE) 0.625, and the Cronbach's alpha 0.705, all of which indicated the questionnaires had relatively good reliability and validity.

### 2.5 Data analysis technique

In this study, SmartPLS (PLS-SEM) software, version 4.0, was used to perform the structural equation modeling. The primary objective of this study was to explore the relationship between PEH, EMO (SMO, EJMO, CMO, AMO, and FMO), and EB in Shanghai junior high school students. The final objective was to develop a framework of PEH, EMO (SMO, EJMO, CMO, AMO, and FMO), and EB for Shanghai junior high school students.

PLS-SEM was chosen as the analysis technique for this study based on several reasons. First, PLS-SEM has the ability to utilize smaller sample sizes compared to other structural modelling techniques (25). Second, PLS-SEM has the advantage of enabling the entire research model to be tested at once (25). In the context of this study, the relationships of the entire research model were tested at once with the loadings of each item and the AVE indicated clearly in the output diagram. Lastly, PLS-SEM shows the evaluation criteria and the significance of every relationship between the independent variables and the dependent variable examined in this study (25). PLS also offers a wide range of statistical indicators and graphical displays to evaluate model fit and parameter estimation (25). Compared to Covariance-Based SEM (CB-SEM), CB-SEM is primarily focused on theory testing, PLS-SEM is more suitable for predictive-causal investigation (26).

## 3 Results

First, the measurement model was assessed to evaluate convergent validity through factor loadings, composite reliability (CR), and the average extracted (AVE) (27). The recommended value of 0.6 or above is set for loadings, 0.7 or above for composite reliability, and 0.5 or above for AVE. Table 1 presents the results of factor loadings, Cronbach's alpha, composite reliability, and AVE for all constructs. The variance inflation factor (VIF) was examined for all items of the included constructs for multicollinearity. According to the results, the absence of multicollinearity is indicated by the estimated VIF values, which does not exceed 5 (Table 1). The VIFs suggested that the model may be evaluated structurally in greater detail, and thus, the requirements for a reflective measurement model may be satisfied.

**Table 1:** Construct reliability and convergent validity.

Construct name	Outer loading		VIF	CA	CR	AVE
Parents’ exercise habits	PEH1	0.830	1.484	0.729	0.847	0.648
	PEH2	0.811	1.450			
	PEH3	0.774	1.387			
Exercise behavior	EB1	0.731	1.383	0.705	0.833	0.625
	EB2	0.825	1.510			
	EB3	0.813	1.308			
competence motivation	CMO 1	0.873	2.177	0.863	0.916	0.785
	CMO 2	0.885	2.222			
	CMO 3	0.899	2.235			
Appearance motivation	AMO1	0.884	2.016	0.850	0.909	0.768
	AMO2	0.899	2.346			
	AMO3	0.845	1.973			
Enjoyment motivation	EJMO 1	0.897	2.880	0.886	0.904	0.814
	EJMO 2	0.947	3.765			
	EJMO 3	0.861	2.163			
Fitness motivation	FMO 1	0.895	2.528	0.893	0.933	0.824
	FMO 2	0.915	2.621			
	FMO 3	0.912	2.913			
Social motivation	MO3	0.825	2.249	0.874	0.919	0.791
	MO7	0.919	2.925			
	MO15	0.921	2.248			

After confirming convergent validity, discriminant validity was assessed using the Fornell–Larcker criterion, cross-loading, and the heterotrait-monotrait (HTMT) ratio of correlation. The results for these three criteria are presented in Tables 2–4, respectively. Using the Fornell-Larcker criterion, discriminant validity was assessed by comparing the square root of each AVE on the diagonal with the correlation coefficients (off-diagonal) for each construct in the relevant rows and columns. As indicated in Table 2, the constructs of PEH, EMO (SMO, EJMO, CMO, AMO, and FMO), and EB exhibited sufficient or satisfactory discriminant validity (28, 29). As indicated in Table 3, every measurement item was loaded higher against its respective latent variables than against other variables. Further, the table below indicated that the loading of each block is higher than that of the other blocks in similar rows and columns. As hypothesized in the conceptual model, the loading clearly separated every latent variable.

**Table 2:** Discriminant validity (Fornell-Larcker's criterion).

Item	CMO	AMO	EB	EMO	FMO	PEH	SMO
CMO	0.886						
AMO	0.585	0.876					
EB	0.313	0.145	0.791				
EJMO	0.800	0.577	0.206	0.902			
FMO	0.723	0.502	0.286	0.564	0.907		
PEH	0.208	0.140	0.422	0.143	0.132	0.805	
SMO	0.746	0.631	0.120	0.772	0.504	0.106	0.889

SMO, social motivation, EJMO, enjoyment motivation; CMO, competence motivation; AMO, appearance motivation; FMO, fitness motivation; PEH, parents’ exercise habits; EB, exercise behavior.

**Table 3:** Loading and cross-loading of measurement items.

Item	CMO	AMO	EB	EJMO	FMO	PEH	SMO
CMO 1	0.873	0.535	0.265	0.761	0.648	0.151	0.653
CMO 2	0.885	0.514	0.273	0.666	0.667	0.182	0.658
CMO 3	0.899	0.509	0.293	0.705	0.611	0.215	0.670
AMO1	0.583	0.884	0.135	0.576	0.442	0.139	0.646
AMO2	0.505	0.899	0.148	0.492	0.434	0.108	0.530
AMO3	0.437	0.845	0.094	0.436	0.450	0.120	0.466
EB1	0.202	0.077	0.731	0.096	0.157	0.235	0.032
EB2	0.222	0.099	0.825	0.124	0.231	0.361	0.094
EB3	0.305	0.156	0.813	0.245	0.272	0.381	0.139
EJMO 1	0.687	0.535	0.159	0.897	0.444	0.141	0.702
EJMO 2	0.755	0.529	0.215	0.947	0.529	0.142	0.710
EJMO 3	0.725	0.502	0.180	0.861	0.555	0.102	0.683
FMO 1	0.674	0.453	0.237	0.515	0.895	0.132	0.459
FMO 2	0.685	0.479	0.289	0.547	0.915	0.125	0.478
FMO 3	0.605	0.433	0.248	0.468	0.912	0.100	0.432
PEH1	0.188	0.114	0.345	0.147	0.132	0.830	0.091
PEH2	0.189	0.147	0.307	0.124	0.118	0.811	0.119
PEH3	0.121	0.073	0.370	0.070	0.064	0.774	0.042
SMO1	0.710	0.571	0.133	0.758	0.495	0.114	0.921
SMO2	0.602	0.493	0.079	0.542	0.419	0.034	0.825
SMO3	0.663	0.608	0.093	0.707	0.423	0.109	0.919

SMO, social motivation; EJMO, enjoyment motivation; CMO, competence motivation; AMO, appearance motivation; FMO, fitness motivation; PEH, parents’ exercise habits; EB, exercise behavior.

**Table 4:** Discriminant validity (HTMT criterion).

Item	CMO	AMO	EB	EMO	FMO	PEH	SMO
CMO							
AMO	0.678						
EB	0.392	0.178					
EJMO	0.918	0.660	0.245				
FMO	0.823	0.577	0.348	0.632			
PEH	0.258	0.175	0.575	0.174	0.161		
SMO	0.848	0.716	0.135	0.853	0.564	0.118	

SMO, social motivation; EJMO, enjoyment motivation; CMO, competence motivation; AMO, appearance motivation; FMO, fitness motivation; PEH, parents’ exercise habits; EB, exercise behavior.

In addition to the Fornell-Larcker and cross-loading criteria, the HTMT criterion was tested to ensure discriminant validity. As indicated in Table 4, the values of the constructs (below 0.90) satisfied the discriminant validity assessment based on the HTMT (30). Hence, the measurement model used in this study has discriminant validity.

To use SEM, the model fit indices indicated an acceptable overall fit. The SRMR value of 0.057 was below the recommended threshold of 0.08, suggesting a good model fit. The d_ULS (0.759) and d_G (0.425) values were within acceptable ranges, indicating limited discrepancies between the observed and model-implied correlations. The NFI value of 0.807 reflected an adequate fit for PLS-SEM. Although the chi-square value (χ^2^ = 2,067.476) was relatively large, this was expected given its sensitivity to sample size.

**Figure 2:**
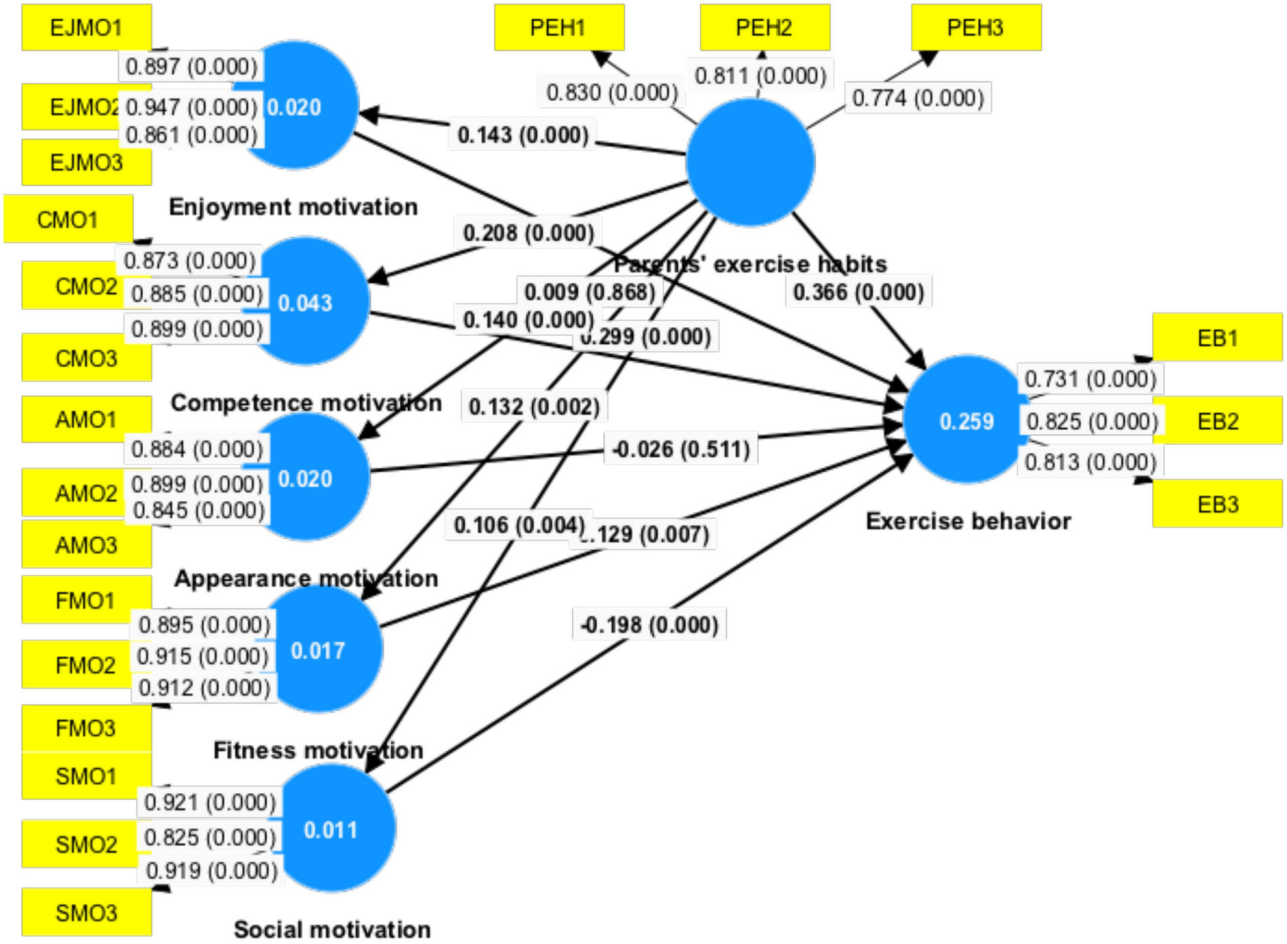
PLS-SEM results.

For path coefficients and *t*-value estimation, this study calculated the standardized beta (*β*) value to assess the importance of the presented hypothesis. When testing for unit-variation in the independent variable PEH, the beta (*β*) value describes the most possible range of dependent-variable constructs to observe the direct effects of PEH and EMO (SMO, EJMO, CMO, AMO, and FMO) constructions toward EB. For the proposed model, we determined a beta (*β*) value for each possible course of action. Higher and significant beta (*β*) levels will have a more extensive influence on endogenous-latent constructs. The *t*-test is a method for assessing the level of significance of the beta (*β*) value. We employed bootstrapping SEM to evaluate the significance of the hypotheses (Table 5; Figure 2).

**Table 5:** Smart PLS-SEM results.

Hypothetical relationships	Beta (*β*)	Standard deviation (STDEV)	T statistics (|O/STDEV|)	*P*-values
H1 = CMO -> EB	0.299	0.069	4.358	0.000**
H2 = AMO -> EB	−0.026	0.040	0.658	0.511
H3 = EJMO -> EB	0.009	0.055	0.166	0.868
H4 = FMO -> EB	0.129	0.048	2.713	0.007**
H5 = SMO -> EB	−0.198	0.056	3.502	0.000**
H6 = PEH -> CMO	0.208	0.038	5.482	0.000**
H7 = PEH -> AMO	0.140	0.037	3.735	0.000**
H8 = PEH -> EJMO	0.143	0.038	3.801	0.000**
H9 = PEH -> FMO	0.132	0.042	3.175	0.002**
H10 = PEH -> SMO	0.106	0.037	2.891	0.004**
H11 = PEH -> EB	0.366	0.040	9.110	0.000**

SMO, social motivation; EJMO, enjoyment motivation; CMO, competence motivation; AMO, appearance motivation; FMO, fitness motivation; PEH, parents’ exercise habits; EB, exercise behavior.

** *p* < 0.01.

As presented in Table 6, the results of H1 (*β* = 0.299, *t* = 4.358, *p* = 0.000) indicated statistical significance at the 1% level for CMO's positive relationship with EB. Consequently, the findings confirm H1, which asserted that CMO had a significance relationship with EB. The results of H4 (*β* = 0.129, *t* = 2.713, *p* = 0.007), H11 (*β* = 0.366, *t* = 9.110, *p* = 0.000), H5 (*β* = −0.198, *t* = 3.502, *p* = 0.000) displayed that FMO were related with EB, PEH had a positive relationship with EB, which was significantly supported. In this research, SMO was related with EB, but their relationship were negative, which was significantly not supported. The results of H6 (*β* = 0.208, *t* = 5.482, *p* = 0.007), H7 (*β* = 0.140, *t* = 3.735, *p* = 0.007), H8 (*β* = 0.143, *t* = 3.801, *p* = 0.007), H9 (*β* = 0.132, *t* = 3.175, *p* = 0.000), H10 (*β* = 0.106, *t* = 2.891, *p* = 0.000) indicated that PEH was positively associated with CMO, AMO, EJMO, FMO, and SMO, which was significantly supported. However, the results of H2 (*β* = −0.026, *t* = 0.658, *p* = 0.511) and H3 (*β* = 0.009, *t* = 0.166, *p* = 0.868), because their *P* values were above 0.05, and thus, AMO had no relationship with EB, and EJMO had no relationship with EB (Figure 2).

**Table 6:** Results of total and specific indirect effects.

Path	Standardized estimate	Standard deviation (STDEV)	T statistics (|O/STDEV|)	*P*-values
Total indirect effects				
PEH -> EB	0.056	0.015	3.781	0.000**
Specific indirect effects				
H12 = PEH -> EJMO -> EB	0.001	0.008	0.157	0.875
H13 = PEH -> SMO-> EB	−0.021	0.010	2.154	0.031*
H14 = PEH -> FMO -> EB	0.017	0.009	1.944	0.052
H15 = PEH -> AMO -> EB	−0.004	0.006	0.618	0.537
H16 = PEH -> CMO -> EB	0.062	0.018	3.444	0.001**

SMO, social motivation; EJMO, enjoyment motivation; CMO, competence motivation; AMO, appearance motivation; FMO, fitness motivation; PEH, parents’ exercise habits; EB, exercise behavior.

* *p* < 0.05.

** *p* < 0.01.

Furthermore, analysis of total indirect path: PEH → EB (estimate = 0.056, *t* = 3.781, *p* = 0.000), analysis of specific indirect effects revealed two significant indirect paths: PEH → SMO → EB (estimate = −0.021, *t* = 2.154, *p* = 0.031), PEH → CMO → EB (estimate = 0.062, *t* = 3.444, *p* = 0.001) (Table 6).

## 4 Discussion

In this research, the investigation into PEH, EMO, and EB of junior high school students in Shanghai sheds light on several crucial findings. Through Structural Equation Modeling (SEM) analysis, this research established that PEH was significantly related with various dimensions of EMO (SMO, EJMO, CMO, AMO, and FMO) and EB. The present findings were consistent with prior research suggesting that parental influence plays a pivotal role in shaping adolescents' health-related behaviors, particularly their participation in PA. Parents often act as salient role models, and their consistent engagement in regular PA tends to foster similar behavioral patterns in their children (16). Moreover, adolescents with physically active parents are more likely to exhibit identified and intrinsic forms of motivation—that is, they engage in PA because they personally value its benefits or derive enjoyment from the activity—rather than being driven by external regulation such as social pressure or rewards (20). This pattern underscored the importance of internalized motivation, which is more strongly associated with sustained behavioral engagement.

Moreover, the findings underscored the importance of considering both the direct and indirect effects of parental modeling on adolescents EB. Specifically, CMO emerged as significant mediators in the relationship between PEH and EB, highlighting the complex interplay between familial influences and individual motivational factors in shaping EB among adolescents. This research was in line with the previous study that Gunnell et al. (31) indicated that changes in competence satisfaction mediated the relationship between autonomous motivation and PA. Furthermore, changes in autonomous motivation through competence satisfaction mediated the relationship between intrinsic goals and PA.

An interesting and theoretically meaningful finding of this study was that SMO was negatively associated with EB, and consequently, the indirect effect of PEH on EB via SMO was also negative. This was completely contrary to previous studies, which suggested that (32) when parents regularly participate in PA, adolescents may begin to associate exercise with social engagement and family interaction, thereby reinforcing socially driven exercise motives. Additionally, parental encouragement of participation in family or group sports may enhance adolescents' perception of exercise as a socially rewarding behavior (33, 34). From a Self-Determination Theory (SDT) perspective, this result could be explained by considering the controlled nature of social motivation (32). SMO, as measured by the MPAM-R, primarily reflected exercise participation driven by external social influences, such as peer approval, social recognition, or pressure to conform. These motives were more closely aligned with external or introjected regulation, which represented less autonomous forms of motivation. SDT posited that controlled motivation might undermine sustained behavioral engagement, particularly when individuals perceived their behavior as driven by external expectations rather than personal choice.

In the context of Chinese junior high school students, strong social motives were also likely to be accompanied by social comparison, fear of negative evaluation, or performance-related pressure, which could have reduced intrinsic interest and enjoyment in PA. When adolescents engaged in exercise mainly to meet social expectations or to avoid social disapproval, such participation tended to be less self-endorsed and more psychologically taxing, ultimately leading to lower overall exercise engagement. This interpretation was consistent with previous research indicating that socially driven or externally regulated motives had neutral or even negative associations with long-term PA participation among adolescents.

Furthermore, although PEH were positively associated with adolescents' SMO, this pathway did not translate into increased EB. This finding suggested that parental modeling might have inadvertently reinforced socially controlled motives—such as exercising to “fit in” or to meet perceived social norms—rather than fostering autonomous motivation. As a result, the mediating effect of SMO became negative. Taken together, these findings highlighted that not all forms of motivation functioned uniformly in promoting EB. Interventions aiming to leverage parental influence therefore needed to emphasize autonomy-supportive practices that nurtured intrinsic and competence-based motivations, rather than relying on social pressure or external social reinforcement.

In this research, EJMO and AMO had no relationship with EB in our study, they were nonetheless influenced by PEH. For EJMO, while theoretically aligned with intrinsic regulation under self-determination theory (19) may not always translate into consistent behavioral patterns, particularly in structured or externally imposed environments such as school settings. Adolescents may report enjoying PA, but still lack the autonomy or opportunity to engage in it regularly. Moreover, enjoyment might be a consequence of repeated engagement in PA rather than a cause, making it less predictive of initial behavioral action. For AMO, a form of external regulation, is often linked with short-term or unstable engagement in PA, especially among adolescents who are still developing body image awareness. While adolescents may express a desire to exercise for appearance-related reasons, such motivation may lack the internalization necessary to sustain regular PA. Furthermore, social pressures and media ideals could create conflicting emotions, leading to exercise avoidance instead of commitment. It is also worth noting that developmental and cultural factors may play a role. In Chinese adolescent populations, academic pressure and limited discretionary time may reduce the salience of intrinsic enjoyment or aesthetic concerns in influencing PA behaviors, while motivations related to social interaction or perceived competence may carry greater weight.

### 4.1 Implication

Based on the present findings, effective interventions to promote adolescents' EB should be implemented jointly at the family and school levels, with a particular focus on fostering autonomous motivation. At the family level, parents should be encouraged to act as consistent and autonomy-supportive role models by engaging in regular PA with their children, emphasizing enjoyment, skill development, and health benefits rather than social comparison or performance outcomes. Parental communication should focus on supporting adolescents’ sense of choice and competence, thereby strengthening competence and fitness motivations. At the school level, physical education programs should be designed to enhance students' perceived competence through developmentally appropriate tasks, progressive skill challenges, and positive feedback, while minimizing excessive social comparison and evaluative pressure. Schools may also integrate health-oriented and mastery-based physical activity programs that highlight personal improvement and well-being rather than external rewards or peer approval. Collectively, these family- and school-based strategies may help shift adolescents' motivation toward more self-determined forms, thereby promoting sustained engagement in PA.

### 4.2 Limitations and directions for further research

Several limitations of this study should be acknowledged. First, the cross-sectional design precluded causal inferences regarding the relationships among PEH, EMO, and EB. Future longitudinal or experimental studies are needed to examine the directionality and dynamic mechanisms of these associations. Second, all variables were assessed using self-reported questionnaires, which may be subject to recall bias and social desirability effects; future research could incorporate objective measures of PA and multi-informant data. Third, the sample was limited to junior high school students in Shanghai, which may restrict the generalizability of the findings to other regions or cultural contexts. Future studies should therefore include more diverse populations and consider additional contextual factors, such as school environments and peer influences, to further clarify the motivational processes underlying adolescents' EB.

## 5 Conclusion

This study examined the relationships among PEH, EMO, and EB among junior high school students in Shanghai within the SDT framework. The results showed that PEH influenced adolescents' EB both directly and indirectly, with competence and fitness motivations acting as key positive mediators, while social motivation exhibited a negative mediating effect. These findings highlighted the distinct roles of autonomous and controlled motivations in shaping EB. The study underscored the importance of promoting autonomy- and competence-supportive environments in both family and school settings to foster sustained PA among adolescents.

## Data Availability

The original contributions presented in the study are included in the article/Supplementary Material, further inquiries can be directed to the corresponding author.
